# The lost generation of *Pemphigus populiglobuli* (Hemiptera, Aphididae): exploring the taxonomy of the Svalbard aphids of genus *Pemphigus*

**DOI:** 10.1186/s40851-024-00245-w

**Published:** 2024-12-18

**Authors:** Karina Wieczorek, Dominik Chłond, Emmanuelle Jousselin, Stephen J. Coulson

**Affiliations:** 1 Institute of Biology, Biotechnology and Environmental Protection, Faculty of Natural Sciences, University of Silesia in Katowice, Bankowa 9, Katowice, 40-007 Poland; 2 UMR 1062 Centre de Biologie pour la Gestion des Populations, INRAe, CIRAD, Institut Agro, IRD, Univ. Montpellier, 755 avenue du Campus Agropolis, Montferrier-sur-Lez, 34988 France; 3 Department of Arctic Biology, University Centre in Svalbard (UNIS), P.O. Box 156, 9171 Longyearbyen, Svalbard Norway

**Keywords:** Arctic, COI, Insect, Overwintering, *Pemphigus groenlandicus*, Taxonomy

## Abstract

Species identification within the aphid genus *Pemphigus* Hartig, 1839 poses challenges due to morphological similarities and host-plant associations. Aphids of this genus generally exhibit complex life cycles involving primary hosts (poplars) and secondary (mostly unrelated herbaceous) host-plants, with some species relying solely on root-feeding generation. An example is a representative of the genus *Pemphigus*, trophically associated with grass roots, found in the High Arctic Svalbard archipelago. Historical records tentatively identify it as *Pemphigus groenlandicus* (Rübsaamen, 1898), although its formal classification remains elusive, due to limited material of freshly collected samples. Recent collections from 2007 to 2024 across various Svalbard sites, revealed its presence under stones in sheltered microhabitats, providing valuable specimens for comparative studies. Our molecular analyses indicate that the Svalbard specimens are not a separate species commonly identified as *P. groenlandicus*, nor an anholocyclic generation of *Pemphigus bursarius* (Linnaeus, 1758) or *P. borealis* Tullgren, 1909, but represent a secondary generation of *Pemphigus populiglobuli* Fitch, 1859, the Nearctic poplar bullet gall aphid. This suggests that they may have lost their primary host associations and adapted to living on grass roots year-round. Our specimens did not host any known facultative symbionts; however, we detected a strain of *Pseudomonas* Migula, 1894, closely related to a cold-tolerant bacterium abundant in polar regions. The present study also investigates the taxonomic relationships and morphometric characteristics of grass-feeding *Pemphigus* populations across the Arctic and an isolated locations on the European continent. Specimens from Svalbard were compared with samples from Greenland and Iceland, but identified no substantial morphometric differences among these geographically separated populations. Similarly, analyses of samples of *Pemphigus groenlandicus crassicornis* Hille Ris Lambers, 1952 from Sweden and Spain reveals a high morphometric similarity to the Arctic population, indicating a strong link between these traits and geographical variability. Despite the limitations in fresh material availability across locations, minor morphometric variations and shared ecological niches (all populations studied inhabiting grass roots, a unique trait within the *Pemphigus* genus) suggest treating both *P. groenlandicus* and its subspecies *crassicornis* as a junior synonym to *P. populiglobuli*. The study also demonstrates that the secondary generation of *P. populiglobuli* is a terrestrial microarthropod that overwinters in a postembryonic life-stage in situ in soil and vegetation under harsh Arctic conditions, and its cryptic life complicates its distribution mapping.

**Supplementary Information:**

The online version contains supplementary material available at 10.1186/s40851-024-00245-w.

## Background

The Svalbard archipelago, situated between 74° to 81° N and 10° to 35° E, is one of the High Arctic regions most extensively studied for its biodiversity, including its invertebrate fauna [1, 2]. While the total number of species is lower than in more temperate regions, there is considerable diversity and endemism within certain groups, particularly among insects. Despite the characteristic High Arctic environment, approximately 260 insect taxa have been documented in Svalbard, with the most numerous orders represented by Diptera, Phthiraptera, Hymenoptera, and Coleoptera [3]. Within this diverse insect community, the order Hemiptera is notably represented by two native species of aphids (Aphididae): *Acyrthosiphon svalbardicum* Heikinheimo, 1968 and *Sitobion calvulum* (Ossiannilsson, 1958), belonging to the Aphidinae subfamily. First described in the mid-20th century, both are holocyclic, monophagous species, associated with the eight-petal mountain-avens *Dryas octopetala* L., and the polar willow *Salix polaris* Wahlenb., respectively [3, 4]. Unlike most aphid species in temperate climates, these endemic Arctic species exhibit an exceptionally short life cycle. Stem mothers produce sexual morphs directly, with few parallel viviparous generations [5, 6]. This genetically controlled, shortened life cycle is adapted to Arctic conditions, including short, cool summers and continuous sunlight during long polar days. The overwintering strategy involves the production of specialized eggs that can withstand freezing temperatures [7]. These aphids exhibit highly restricted distributions, known only from a few scattered localities across Spitsbergen, the largest island in the Svalbard archipelago [8]. Both *A. svalbardicum* and *S. calvulum* are considered globally rare and endemic to Svalbard, highlighting their unique evolutionary history and adaptation to the Arctic conditions. Their exceptional biological traits have positioned them as model species within numerous studies exploring the life strategies and ecology of terrestrial arthropods in the Arctic environment [9–13].

The third and least known among the Svalbard aphids is an unidentified species belonging to the genus *Pemphigus* Hartig (Eriosomatinae). This species was initially referred to as *P. borealis* Tullgren and documented by Thor [14], when it was collected under boards in grass and moss in June 1928, near Barentsburg. Subsequently, in August 1933, similar specimens associated with grass and identified as *Pemphigus* sp. were recorded in two locations – the area of Tempelfjorden and in Longyearbyen (slides from NHMUK, London, UK). All of these locations were situated on Spitsbergen. Heikinheimo [4] recognized material collected by Thor as probably congeneric with *P. groenlandicus* (Rübsaamen, 1898) s. str., which was newly re-described by Hille Ris Lambers from Greenland [15], who also established its subspecies *crassicornis*. The species was originally described by Rübsaamen [16] as *Tychea groenlandica*, found on grass roots in the Uummannaq fiord area of Greenland. Mordvilko [17], in turn, identified similar material from grass roots collected on Iceland as *P. bursarius* (L.), a classification rejected by Hille Ris Lambers [18], who instead identified this species also as *P*. *groenlandicus* s. str. Subsequent authors primarily used this name in inventory works on Svalbard fauna [19, 20]. However, this taxon requires formal identification, which is challenging as, until the first decade of the present century, additional aphid material from Svalbard was practically unknown and the samples collected by Sig Thor were lost.

Another complicating factor arises from the complex life cycles exhibited by *Pemphigus* aphids. The genus *Pemphigus* encompasses over 70 species, widespread throughout the northern Hemisphere [21]. The most common form of the life cycle in aphids of this genus is heteroecy, which involves a change in the host plant. The primary host of *Pemphigus* are various species of *Populus* spp. (cottonwood or poplar trees), in which spring females of viviparous generation (fundatrices) form distinctive galls on leaves or twigs of the host plants. In early summer their winged progeny (fundatrigeniae) migrate to various herbaceous secondary hosts, primarily belonging to families such as Amaranthaceae, Apiaceae, Brassicaceae, Compositae/Asteraceae, Euphorbiaceae, Leguminosae/Fabaceae, and Ranunculaceae, exceptionally to Poaceae [22]. These aphids establish colonies on the roots or occasionally form woolly wax masses above soil level. On the secondary host, only parthenogenetic generation (wingless viviparous females) occur. In early autumn, low temperatures induce the return migration of winged sexuparae to the primary host. Sexuparae produced on the bark crevices dwarfish males and oviparous females, which, after mating deposit a single overwintering egg [23, 24]. However, the complete life cycle is known for only about 16 species of the genus *Pemphigus*. For the vast majority of aphids of this genus only the gall-inducing generation on the primary host has been described and many species are distinguished primarily based on the morphology of galls. About ten species, including *P*. *groenlandicus*, are known solely from their root-feeding generation, sharing many morphological characteristics. Thus, identification is often difficult, or even impossible, and relying solely on wingless viviparous generation for species-level host records is of dubious utility. Within holocyclic, host-alternating species, some have a life cycle that extends two years (e.g., *P. mordwilkoi* Cholodkovsky, 1912), while some species are capable of anholocyclic overwintering on roots of secondary hosts (e.g., *P. betae* Doane, 1900, *P. bursarius* (Linnaeus, 1758), *P. populinigrae* (Schrank, 1801), *P. trehernei* Foster, 1976), or sexuparae overwintering on roots of secondary hosts, and eggs are deposited in early spring on primary hosts for a very short period of hatching (e.g., *P. obesinymphae* Aoki and Moran, 1994). Four species are holocyclic and monoecious on *Populus* spp., with winged sexuparae produced within the galls, whereas one species is likely permanently anholocyclic, feeding on *Salix* spp. or *Vitis vinifera* roots (*P. saliciradicis* (Börner, 1950)) [22].

Given the diverse life cycles observed among aphids in the *Pemphigus* genus, coupled with limited research and sparse documentation regarding the grass root-feeding population of Svalbard aphids within this genus, their taxonomic position, biology, and ecology, remain poorly understood. Using (limited) fresh material of grass-rooting individuals of the genus *Pemphigus* from the Arctic region, we sequenced the DNA gene fragment used in aphid barcoding studies [25] to test whether Svalbard populations of this genus belong to any known species of *Pemphigus* referenced in Barcode databases. As some previous authors have identified individuals of the genus *Pemphigus* found on Svalbard as corresponding to *P. groenlandicus* [4, 18], we further conducted comparative morphometric studies on the available material of grass-rooting individuals from Svalbard, Iceland, Greenland, Sweden, and Spain, labelled as *Pemphigus* sp., *P. groenlandicus*, or *P. groenlandicus* subsp. *crassicornis.* We also provide new data on the morphology, biology and distribution of the Svalbard population studied as well as its microbiome profile. By investigating the grass root-feeding population of Svalbard aphids within this genus, we can contribute to a broader understanding of aphid life cycle variations. With this study, we aim to fill these knowledge gaps and provide valuable insights into the taxonomic position, biology, and ecology of these aphids, particularly in harsh Arctic conditions. As the Arctic undergoes rapid environmental changes due to the global warming, understanding how aphids adapt and thrive in these conditions is essential. This study may provide insights into the resilience of aphids to environmental stressors and their potential responses to ongoing climate change.

## Materials and methods

### Sample collection, fixation and storage

Fresh material—adult wingless viviparous females and their nymphs—were hand collected with a pooter (an aspirator) in two locations: (1) from under stones, behind a disused cattle and pig shed, 78.217° N 15.616° E, on 31 July 2021 (summer population) and (2) from roots of *Poa* sp. tussocks sampled from partly frozen ground from behind the University Centre in Svalbard (UNIS) 78.222° N 15.653° E, on 27 September 2023, 10 October 2023, and 28 May 2024 (hibernating population), both sites in Longyearbyen, Svalbard, Norway. The aphids were obtained from the *Poa* sp. tussocks by gentle extraction in a Tullgren funnel system (Burkard Scientific Ltd., Uxbridge, U.K.) into 96% ethanol or distilled water at UNIS. From every location at least 30 individuals were fixed in both 70% ethanol, 96% ethanol, and 2.5% glutaraldehyde in 0.1 M phosphate buffer (pH 7.4) for further molecular and morphological study. Voucher specimens corresponding to the extracted individuals were slide-mounted and deposited in the entomological collection of the University of Silesia in Katowice, Poland (DZUS).

### Barcoding COI DNA sequences and analyses

DNA was extracted from three specimens from the summer and hibernating populations respectively. They were left in lysis buffer overnight from a DNA Qiagen extraction kit; the extraction protocol then followed the manufacturer’s instructions, except that the final elution volume was 70 µl. We conducted a non-destructive DNA extraction as advised by Favret [26]. Primers used for amplifying the mitochondrial cytochrome c oxidase subunit I (COI) gene fragment usually used for Barcoding Aphididae were LepF (5′-ATTCAACCAATCATAAAGATATTGG-3′) (forward) and LepR (5′-TAAACTTCTGGATGTCCAAAAAATCA-3′)′ [25]. The PCR mixture included 12.5 µL of Master Mix with 1.25 µL of each primer (10 µM), 7 µL of nuclease-free water and 2 µL of DNA template. Amplification included 30 s denaturation at 98 °C followed by 35 cycles each consisting of 30 s denaturation at 98 °C, 30 s of annealing at temperature of 48 °C and 1 min extension at 72 °C. A final extension was carried out at 72 °C for 5 min. PCR products were electrophoresed on 1% agarose to check for PCR success. Amplicons were then sequenced by Sanger Sequencing using ABI Prism 3500 (Applied Biosystems, CA, USA). Electropherograms were corrected and a consensus of forward and reverse sequence was done using Geneious V 11 (https://www.geneious.com). The three COI sequences obtained have been submitted to National Center for Biotechnology Information NCBI (accession numbers PQ139145, PQ139146, PQ139147).

All sequences of aphids from the *Pemphigus* genus available in BOLD (https://www.boldsystems.org/ last accessed 12/02/2024) database were downloaded. We built two datasets: one dataset including all COI sequences of at least 600 bp, and a second including only sequences that were identified to the species level.

For each dataset, sequences were aligned using Mafft v.7.2.2.1 [27]. We then chose the best model using ModelFinder as implemented in IQ-TREE v2.1.3 (option MF) and ran a Maximum Likelihood (ML) analysis using the best fitting model and 1000 ultrafast bootstrap.

### Endosymbiont characterization

Using the same DNA extractions as for COI sequencing, we applied a protocol for characterizing endosymbionts associated with previously studied aphid samples [28]. Briefly, a 251 bp portion of the V4 region of the 16 S rRNA gene was amplified using primers and the dual-index sequencing strategy developed by Kozich et al. [29]. Each DNA extract was amplified along with negative controls (DNA extraction and PCR controls). These PCR products were then pooled with samples from other microbiome studies, purified and quantified with the Kapa Library Quantification Kit (Kapa Biosystems). The DNA pool was then paired-end sequenced on an Illumina MiSeq flowcell with a 500-cycle Reagent Kit v2 (Illumina). Sequencing results were first filtered through Illumina’s quality control procedure. We then used FLASH v1.2.11 [30] to merge paired sequences into contigs and CUTADAPT v1.9.1 [31] to trim primers. The FROGS pipeline [32] was then used to generate an abundance table of symbiont lineages across samples. Briefly, we first filtered out sequences *>* 261 and *<* 241 bp and clustered variants with Swarm [33] using a maximum aggregation distance of 1. Lastly, we removed chimeric variants with VSEARCH [34]. Taxonomic assignments of clusters was carried out using RDPtools v2.0.3 and BLASTn + against the Silva database release 138 [35]. We also used the webtool leBIBI IV, which provides automatic phylogenetic placement of bacterial 16S sequences [36]. Following taxonomic affiliation, we aggregated clusters when they shared the same taxonomy with at least 98% of identity. From the abundance table, we transformed read numbers per aphid sample into percentages. As endosymbiont abundance cannot reliably be estimated using this method [28], we only discuss the presence/absence of symbionts.

### Morphological analyses

For comparative morphological study, about 30 adult wingless viviparous females from the summer and hibernating populations were initially examined and photographed using a Nikon SMZ 25 stereo microscope with a DSRI2 camera and then slide-mounted using the method of Wieczorek [37].

Additionally, we examined 33 slides including about 33 mounted adult wingless viviparous females and five winged viviparous females (sexuparae) of grass-rooting individuals from Svalbard, Iceland, Greenland, Sweden, and Spain, labelled as *Pemphigus* sp., *P*. *groenlandicus* or *P*. *groenlandicus* subsp. *crassicornis*, including the type material for the latter. The material was loaned from the Natural History Museum, London, UK (NHMUK) and the Muséum national d’histoire naturelle, Paris, France (MNHN). All slide-mounted material was examined and photographed using a Nikon Ni-U light microscope equipped with a phase contrast system and a DS-Fi2 camera. The measurements were taken according to Ilharco & van Harten [38] using a Nikon NIS Elements D 4.50.00 64-Bit software and presented in millimeters (mm). The figures were prepared using Corel Draw ver. 23 (Corel Corporation, Ottawa, Ontario, Canada).

### Scanning electron microscopy

Specimens for SEM analysis (10 adult wingless viviparous females from the summer and hibernating populations) were preserved in 70% ethanol or 2.5% glutaraldehyde. Dehydration of preserved samples was provided by ethanol series of 80%, 90%, 96% and then of two changes of absolute as follows: 20 min in 80% ethanol, 15 min in 90% ethanol, 10 min in 96% ethanol, and two baths in absolute ethanol of 10 min each. Specimens examined by this method will be referred to as ‘waxed,’ since most or all of their waxy secretions were still intact and in their natural position ‘in situ’.

In the second method, used for studying the wax producing structures (wax plates and pores) on the cuticle surface, aphid specimen were ‘dewaxed’, i.e. cleaned from their natural waxy secretions by a method modified from that described by Lucchi & Mazzon [39], i.e. 20 min in 80% ethanol, 15 min in 90% ethanol, 10 min in 96% ethanol, two baths in proportion of 50:50 absolute ethanol and 100% chloroform, 15 min each. The specimen were then kept in 100% chloroform for 48 h at room temperature in a fume hood. Finally the samples were kept by two baths in proportion of 50:50 absolute ethanol and 100% chloroform, 15 min each and two baths in absolute ethanol, 10 min each.

Samples of adult wingless viviparous females from both methods were dried in a Leica EM CPD300 critical point dryer (Leica Microsystems, Vienna, Austria). Samples were mounted on aluminium stubs with double-sided adhesive carbon tape and sputter-coated with a 30-nm layer of gold using a Safematic CCU-010 HV coating unit (Safematic GmbH, Zizers, Switzerland). Coated samples were imaged with a Hitachi SU8010 field emission scanning electron microscope (FE-SEM, Hitachi High-Technologies Corporation, Tokyo, Japan) at 7.0 and 10.0 kV accelerating voltage with a secondary electron detector.

## Results

### DNA barcoding

In preparation for the present study, 1421 sequences of *Pemphigus* were retrieved from the BOLD database. Once we discarded sequences that were of insufficient length or that covered a COI fragment that did not overlap with those of interest in this study, we were left with a dataset of 813 sequences. Once we excluded sequences that were not identified to species, we ended up with a dataset of 434 COI sequences.

**Fig. 1:**
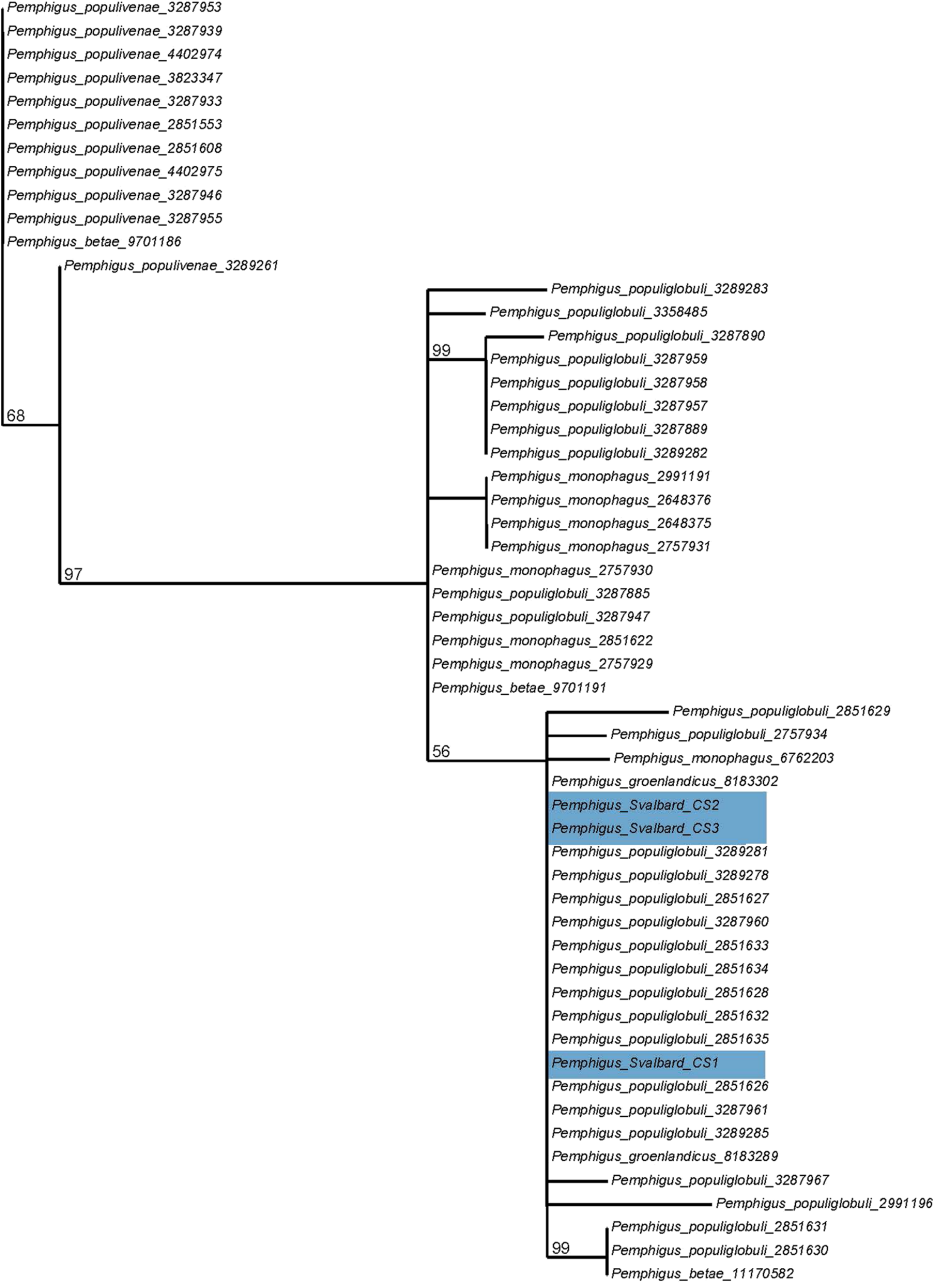
Extraction of the COI ML tree showing the clade including the Svalbard specimens sequenced from this study (colored in light blue) and previous specimens identified as *P. groenlandicus* present in Bold Systems

For both datasets, TIM2 + F + R was identified as the best fitting model of evolution. The ML trees placed our specimen (Pemphigus_Svalbard_CS 1 to 3) in a clade composed of specimens identified as *Pemphigus populiglobuli* Fitch, two specimens from Svalbard identified as *Pemphigus groenlandicus* and four specimens identified as *Pemphigus monophagus* Maxson, 1934 (Fig. 1). Our sequence had a 100% identity with sequences of specimens assigned to *P. populiglobuli* and *P. groenlandicus* in BOLD. According to data from the BOLD database, *P. populiglobuli* were collected on *Populus balsamifera* and some unidentified *Populus* trees, in Alberta, Canada and Utah, USA. The specimens identified as *P. groenlandicus* were collected from Svalbard (Fjortendejulibukta) by S.J. Coulson, with no host plant association. *Pemphigus monophagus* specimens were collected on *Populus balsamifera* in several provinces of east Canada.

Specimens identified as *Pemphigus bursarius* (Linnaeus) or *Pemphigus borealis* Tullgren are all found in well differentiated clusters in the COI ML tree (Additional file 1). This suggests that *Pemphigus* specimens collected from Svalbard, from three different locations and from the summer and hibernating populations, are not secondary generations of these species nor their anholocyclic forms. Our molecular analyses suggest that it is instead a secondary generation from *P. populiglobuli* (the poplar bullet gall aphid), living on grass roots. Those might have lost primary host associations and live on grass roots year-round .

### Endosymbionts

High-throughput sequencing of the three Svalbard specimens uncovered little diversity in the aphid microbiota. One cluster that accounted for 62% of all sequencing reads was assigned to *Buchnera aphidicola*, as expected. In two of the three samples collected (*Pemphigus* CS2 and *Pemphigus* CS3), we also retrieved a cluster that was assigned to the genus *Pseudomonas* and closely related to *Pseudomonas prosekii.* No known aphid facultative endosymbiont was found. Other identified bacterial taxa represented by very few sequencing reads were ubiquitous bacteria that can also be found in the control sample (i.e. the PCR control). Complete composition of the microbiota (in relative read abundance) is available in Table S1.

### Taxonomy

Family Aphididae Latreille, 1802.

Subfamily Eriosomatinae Kirkaldy, 1905.

Tribe Pemphigini Herrich-Schaeffer, 1854.

Genus *Pemphigus* Hartig, 1839.

Species *Pemphigus populiglobuli* Fitch, 1859.

Fitch. 1859[1858]. Transactions of the New York State Agricultural Society 17:850 [40].

*Tychea groenlandica* Rübsaamen, 1898 Bibl. Zool. (Stuttg.), 20(4):115 [16].

*Pemphigus groenlandicus groenlandicus* (Rübsaamen) Hille Ris Lambers, 1952 Medd. Grønl., 136(1):28 [15] syn. nov.

*Pemphigus groenlandicus crassicornis* Hille Ris Lambers, 1952 Medd. Grønl., 136(1):30 [15] syn. nov.

### Material examined

SA02-453-09-001 Pemphigus sp. Fjortendejulibukta, Svalbard, Norway, 6.VIII.2009, 4 apt. viv. fem., SJ Coulson leg. DZUS.; SA02-453-09-002 Pemphigus sp. Longyearbyen, Svalbard, Norway, 27.VII.2021, 1 apt. viv. fem., SJ Coulson leg. DZUS; SA02-453-09-003 Pemphigus sp. Longyearbyen, Svalbard, Norway, 27.VII.2021, 3 apt. viv. fem., SJ Coulson leg. DZUS; SA02-453-09-004 Pemphigus sp. Longyearbyen, Svalbard, Norway, 27.VII.2021, 3 apt. viv. fem., SJ Coulson leg. DZUS; SA02-453-09-005 Pemphigus sp. Longyearbyen, Svalbard, Norway, 27.IX.2023, Poa sp., 1 apt. viv. fem., SJ Coulson leg. DZUS; SA02-453-09-006 Pemphigus sp. Longyearbyen, Svalbard, Norway, 27.IX.2023, Poa sp., 3 apt. viv. fem., SJ Coulson leg. DZUS; SA02-453-09-007 Pemphigus sp. Longyearbyen, Svalbard, Norway, 27.IX.2023, Poa sp., 3 apt. viv. fem., SJ Coulson leg. DZUS; SA02-453-09-008 Pemphigus sp. Longyearbyen, Svalbard, Norway, 16.X.2023, Poa sp., 1 apt. viv. fem., SJ Coulson leg. DZUS; SA02-453-09-009 Pemphigus sp. Longyearbyen, Svalbard, Norway, 16.X.2023, Poa sp., 4 apt. viv. fem., SJ Coulson leg. DZUS; SA02-453-09-010 Pemphigus sp. Longyearbyen, Svalbard, Norway, 16.X.2023, Poa sp., 4 apt. viv. fem., SJ Coulson leg. DZUS; 010182802 Pemphigus groenlandicus Fjortendejulibukta, Svalbard, Norway, 6.VIII.2009, 4 apt. viv. fem., 1 al. viv. fem., SJ Coulson leg. NHMUK; 010182611 Pemphigus groenlandicus Kapisidiglit, Greenland, 16.VIII.1950, grass, 3 apt. viv. fem., D.H.R.L det., NHMUK; 010182612 Pemphigus groenlandicus Kapisidiglit, Greenland, 27.VII.1950, grass roots, 2 apt. viv. fem., 1 al. viv. fem., D.H.R.L det., NHMUK; 010182596 Pemphigus groenlandicus Akureyri, Iceland, 30.IX.1975, Populus nigra ex Agrostis stolonifera (ex cuie), 2 al. viv. fem., HLGS det., NHMUK; 010182597 Pemphigus groenlandicus Vikurbakki Field Station, Iceland, 8.VIII.1975, Agrostis stolonifera (roots), 4 apt. viv. fem., RNBP det., NHMUK; 010182585 Pemphigus groenlandicus Vikurbakki Field Station, Iceland, 8.VIII.1975, Agrostis stolonifera (roots), 4 apt. viv. fem., RNBP det., NHMUK; 010182613 Cotype Pemphigus groenlandicus subsp. crassicornis Kapisidiglit, Greenland, 30.VII.1950, grass roots, 2 apt. viv. fem., 3 nymphs, D.H.R.L det., NHMUK; 010182614 Cotype Pemphigus groenlandicus subsp. crassicornis Egedesminde, Greenland, 9.VI.1949, grass roots, 6 apt. viv. fem., D.H.R.L det., NHMUK; 010182615 Cotype Pemphigus groenlandicus subsp. crassicornis Kapisidiglit, Greenland, 27.VII.1950, grass roots, 1 al. viv. fem., D.H.R.L det., NHMUK; 2500 Pemphigus groenlandicus subsp. crassicornis Sierra Nevada 2400 m, Espagne, 16.VII.1954, Gramines, 3 apt. viv. fem., Janetschek leg., MNHN; 2501 Pemphigus groenlandicus subsp. crassicornis Suede, Lac N d’Abisco, 20.VIII.1948, Gramines, 5 apt. viv. fem., Balachovsky leg., MNHN; 2502 Pemphigus groenlandicus subsp. crassicornis Lac N d’Abisco, Suede, 20.VIII.1948, Gramines, 4 apt. viv. fem., Balachovsky leg., MNHN.

### Re-description

Wingless viviparous female on secondary host (*n* = 30).

**Fig. 2:**
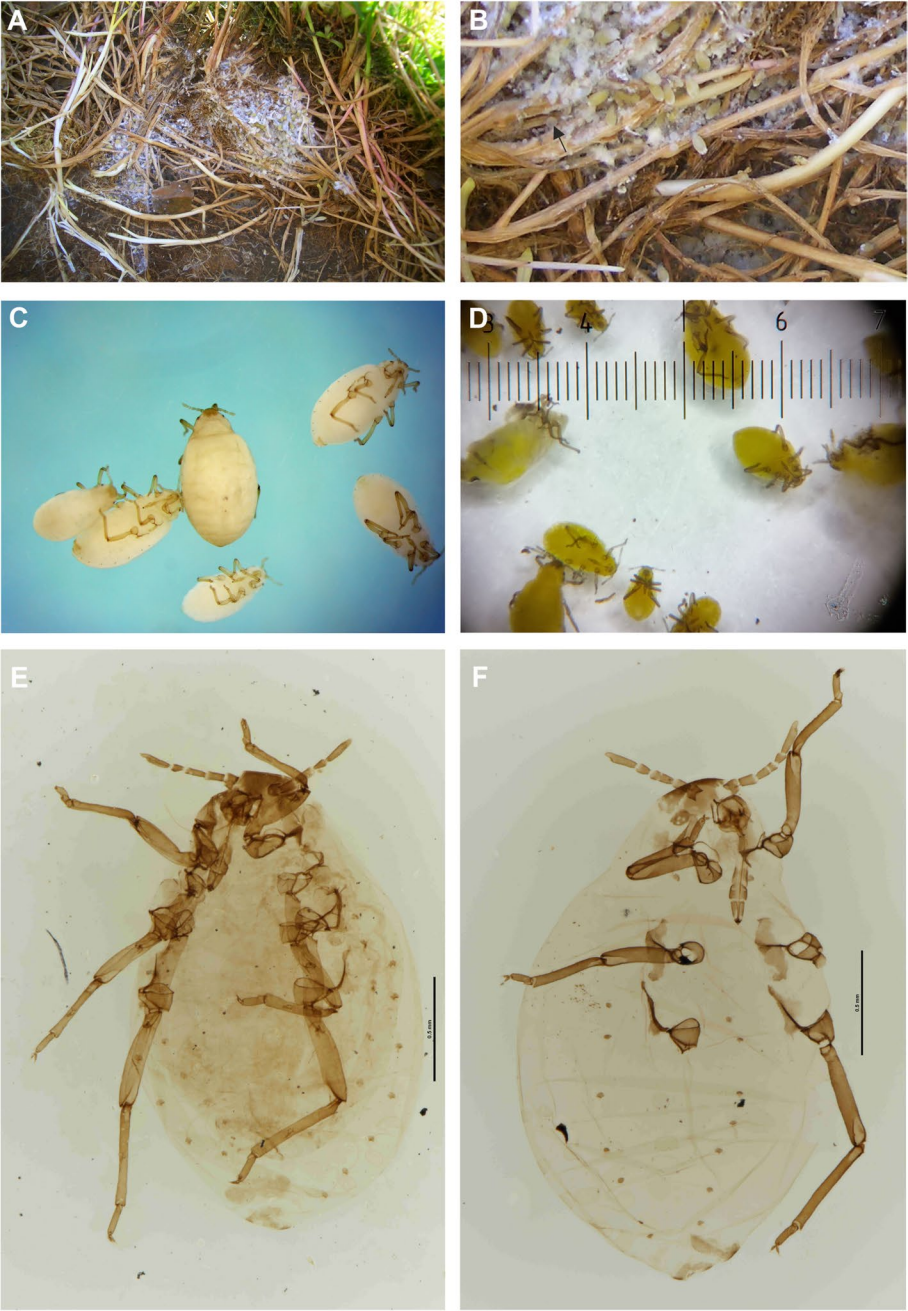
*Pemphigus populiglobuli* secondary generation detected in Svalbard. **A** The waxy colony from beneath flat stones in the lower section of the Fjortendejulibukta bird cliffs. **B** Waxy wingless viviparous female with white tufts on the posterior part of the abdomen; within the wax secretion honeydew droplets coated with wax, forming liquid ‘marbles’ are also visible (arrow). Fresh specimens of summer **C** and hibernating **D** populations collected in Longyearbyen. Slide-mounted specimens of summer **E** and hibernating **F** populations collected in Longyearbyen

**Fig. 3:**
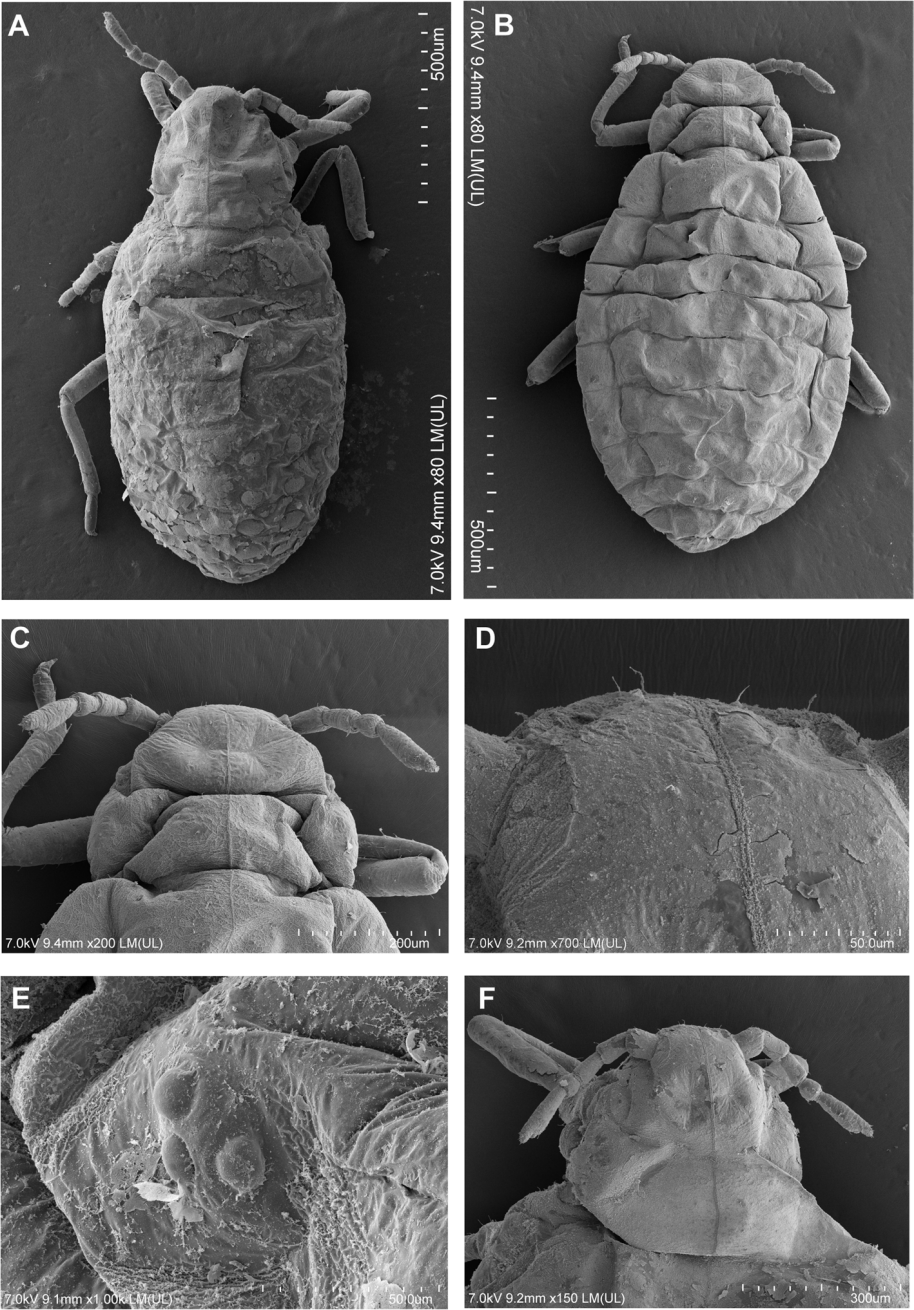
Scanning electron microscopy (SEM) of general morphology of *Pemphigus populiglobuli* secondary generation detected in Svalbard **A** Waxed specimen of the summer population. **B** ‘Dewaxed’ specimen of the hibernating population. **C** Dorsal side of the head, prothorax and mesothorax of ‘dewaxed’ specimen of the hibernating population. **D** Dorsal side of the head of waxed specimen of the summer population. **E** Triommatidium of ‘dewaxed’ specimen of the summer population. **F** Dorsal side of the head, prothorax and mesothorax of waxed specimen of the summer population; wingless viviparous females all collected in Longyearbyen

**Fig. 4:**
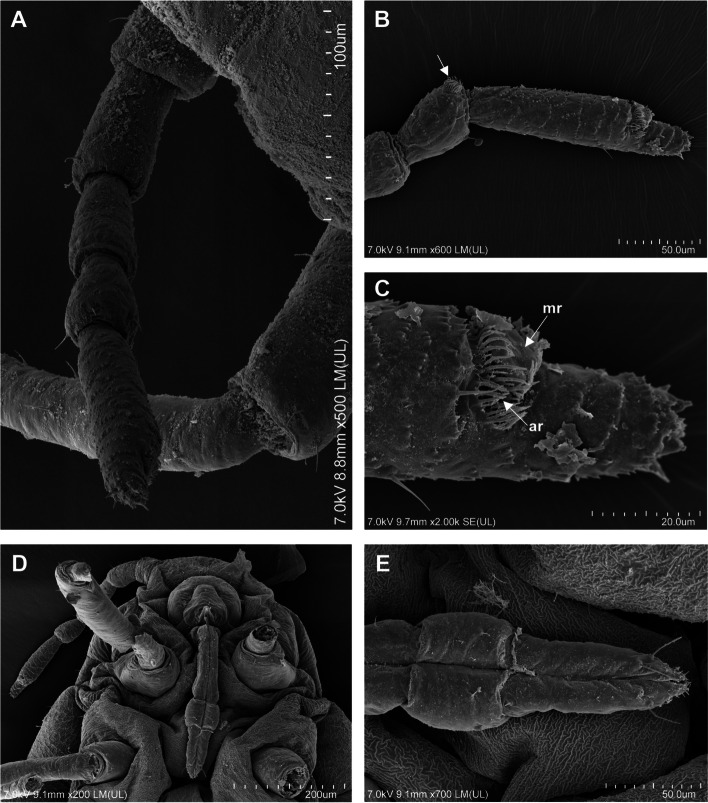
Scanning electron microscopy (SEM) of the general morphology of *Pemphigus populiglobuli* secondary generation detected in Svalbard. **A** Antenna of waxed specimen of the hibernating population. **B** Apical part of the antennal segment IV with big multiporous placoid sensillum with ciliated cuticle edge (arrow) of ‘dewaxed’ specimen of the hibernating population. **C** Base of the antennal segment V with one big multiporous placoid sensillum (mr - major rhinarium) and 1–2 poorly visible small multiporous placoid sensilla (ar - accessory rhinaria) with ciliated cuticle edges of ‘dewaxed’ specimen of the hibernating population. **D** Ventral side of the head and thorax of ‘dewaxed’ specimen of the hibernating population. **E** Ultimate rostral segment of ‘dewaxed’ specimen of the hibernating population; wingless viviparous females all collected in Longyearbyen

**Fig. 5:**
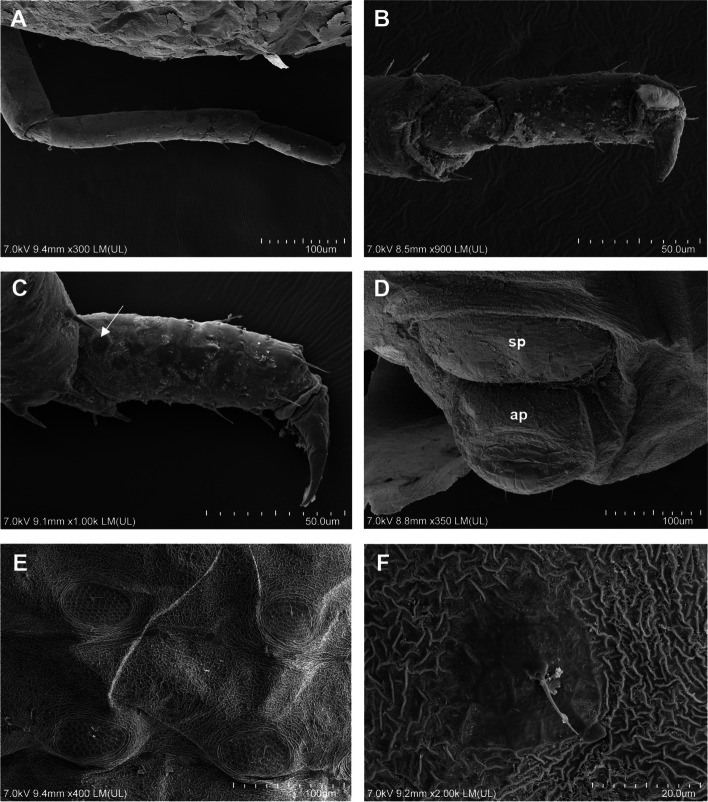
Scanning electron microscopy (SEM) of the general morphology of *Pemphigus populiglobuli* secondary generation detected in Svalbard. **A** Hind legs of waxed specimen of the summer population. **B** Hind tibia and tarsus of ‘dewaxed’ specimen of the summer population. **C** Hind tarsus of ‘dewaxed’ specimen of the summer population with visible campaniform sensillum (arrow). **D** Ventral side of posterior part of the abdomen of waxed specimen of the summer population with visible subgenital plate (sp) and anal plate (ap). **E** Dorsal side of the posterior part of the abdomen of ‘dewaxed’ specimen of the hibernating population with clear wrinkled ornamentation of the cuticle. **F** Smooth area of the cuticle at the base of the abdominal marginal seta of ‘dewaxed’ specimen of the hibernating population; wingless viviparous females all collected in Longyearbyen

**Fig. 6:**
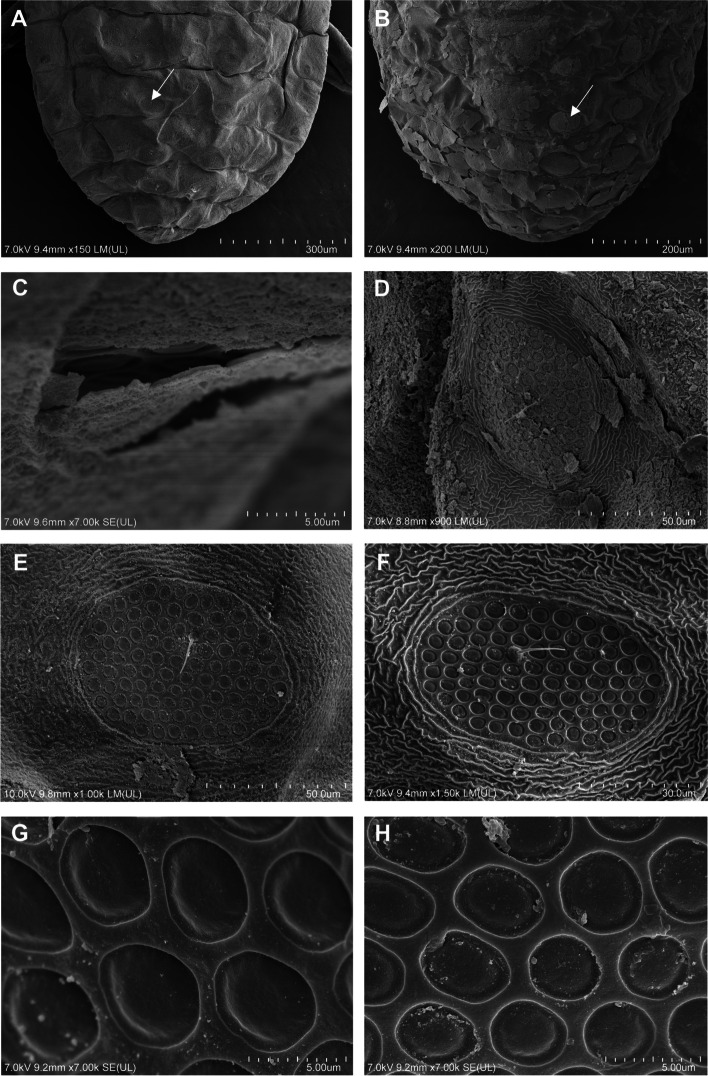
Scanning electron microscopy (SEM) of the general morphology of *Pemphigus populiglobuli* secondary generation detected in Svalbard. Dorsal side of the abdomen with wax gland plates (arrow) of **A** ‘dewaxed’ specimen of the hibernating population and **B** of waxed specimen of the summer population. **C** Layer of wax visible above the gland plate of waxed specimen of the summer population. Wax gland plates visibility depending on the reagent used for the specimens fixations **D** in 70% ethanol, **E** in 2.5% glutaraldehyde, **F** in 2.5% glutaraldehyde and ‘dewaxed’ using chloroform. Wax pores of **G** ‘dewaxed’ specimen of the summer population and **H** of waxed specimen of the hibernating population; wingless viviparous females all collected in Longyearbyen

Figures 2, 3, 4, 5 and 6; Table S2.

Color in life: yellowish, thickly covered with white wax (Fig. 2A), which also forms tufts on the posterior part of abdomen (Fig. 2B) with head, antennae, mouthparts and legs distinctly dark brown.

Coloration of fresh fixed specimen in 70% or 96% ethanol or 2.5% glutaraldehyde: summer forms lemon yellow without visible wax layer (Fig. 2C). Hibernating form more greenish than yellow (Fig. 2D).

Pigmentation of cleared specimens on slides: thorax and abdomen yellowish, head, antennae, mouthparts, spiracles and legs dark brown, wax, subgenital and anal plates dusky (Fig. 2E-F).

Morphometric characters: body egg shaped (Fig. 3A), in ‘dewaxed’ specimens (cleaned from their natural waxy secretions), the segmentation of the body is clearly visible (Fig. 3B). Head not fused with prothorax. Epicranial suture developed (Fig. 3C). Frons flat. Median margin of head with two pairs of very short, pointed setae (Fig. 3D). Triommatidia are the only eyes (Fig. 3E). However, these characters are poorly visible in “waxed” specimens (Fig. 3F). Antennae usually 5-segmented, rarely 6-segmented (which may result from incomplete division of the antennal segment III, Fig. 2F), 0.19–0.23 × body length. Antennal segments II and III of similar length; antennal segment IV distinctly shorter than antennal segment V; antennal segment V the longest; processus terminalis very short, 0.27–0.36 × base; other antennal ratios: V:III 1.40–1.86, IV:III 0.40–0.60. Surface of antennal segments corrugate. Antennae covered with pointed, short, colourless setae that are never longer than the basal articular diameter of antennal segment III. ANT I with 3–4 setae, ANT II with 2–3 setae, ANT III with 0–1 setae, ANT IV with 2–4 setae, base of antennal segment V with two setae (type I trichoid sensilla), processus terminalis with six spine-like apical setae (type II trichoid sensilla Fig. 4A). Apical part of antennal segment IV with one primary rhinarium (big multiporous placoid sensillum with ciliated cuticle edge, Fig. 4B). Base of antennal segment V with one primary (major) rhinarium (big multiporous placoid sensillum with ciliated cuticle edge) and 1–2 poorly visible accessory rhinaria (small multiporous placoid sensilla Fig. 4C). Rostrum reaching to middle coxae (Fig. 4D). Ultimate rostral segment with convex sides, tapering gradually to an acute apex, with three pairs of primary setae and lack of accessory setae (Fig. 4E) 0.70–1.28 × antennal segment III, 0.62–0.83 × second segment of hind tarsus. Legs normal, tibiae scattered with rows of short setae, distributed mainly on their external edges (Fig. 5A) and two pairs of spine-like setae on the apex (Fig. 5B). Tarsi are two-segmented. First tarsal segments with 3:3:2 setae. The proximal part of the second segment of the tarsus bears one rounded campaniform sensillum and ventrally is slightly spinulose. The claws are normal-shaped (Fig. 5C). Siphuncular pores absent. Subgenital plate rounded with a narrowing in the middle, its anterior margin with four setae, posterior margin with 6–8 setae. Anal plate with six setae. Cauda slightly visible, with two setae (Fig. 5D). In ‘dewaxed’ specimens wrinkled ornamentation of the cuticle is visible (Figs. 4D and 5E), with exception of smooth areas at the base of thoracic and abdominal marginal setae (Fig. 5F).

Distribution and ultrastructure of wax gland plates: wax gland plates are present on dorsal abdominal segments III-VII: four plates on each of the segment III-VI and two plates on segment VII in pleural and spinal position (Fig. 6A). In “waxed” specimens the secretion of the glands covers the entire body of the insect (Figs. 3A and 6B) in the form of about 300 nm thick layer (Fig. 6C). Depending on the reagent used to fix the aphid, the wax remains on the surface of the glandular plate (and the entire body of the insect) to varying degrees. 70% ethanol only slightly removed the wax (Fig. 6D), 2.5% glutaraldehyde removed the wax to a significant extent (Fig. 6E), while chloroform removed the wax secretion most effectively, especially after prior fixation with 2.5% glutaraldehyde (Fig. 6F). The single oval wax gland plate is built with wax pores distributed around single dorsal seta measuring 9.9–14.4 μm in length (Fig. 6D-F). The average size of the wax gland plates are 38.0 × 80.5 μm. Wax gland plates on the dorsal abdominal segment III are the smallest, whereas those on the abdominal segment VII the largest. Plates on segments III-VI have approximately 84–98 pores, while plates on segment VII have approximately 120 pores. The wax pores are rather uniform, rounded, with an average diameter 5.5 μm. Each wax pore has a slightly raised rim (Fig. 6G), and a depression in the middle from which wax sheets are exuded (Fig. 6H). Around wax gland plates the dorsal body cuticle forming a distinct sculpture—a rim with thick edges surrounding the plates and wrinkled ornamentation between individual wax gland plates (Fig. 6D–F). There are no significant difference in the size of wax gland plates and number of pores between the summer and hibernating generations of the studied aphid specimens.

*Pemphigus populiglobuli* wingless viviparous females collected from the field site at Longyearbyen in July 2021 were similar in morphology to those found in the second Longyearbyen field site in September/October 2023, but being more greenish than yellow in colour than the summer forms.

Winged viviparous female (sexupara) on secondary host (*n* = 1).

Figure 7; Table S2.

**Fig. 7:**
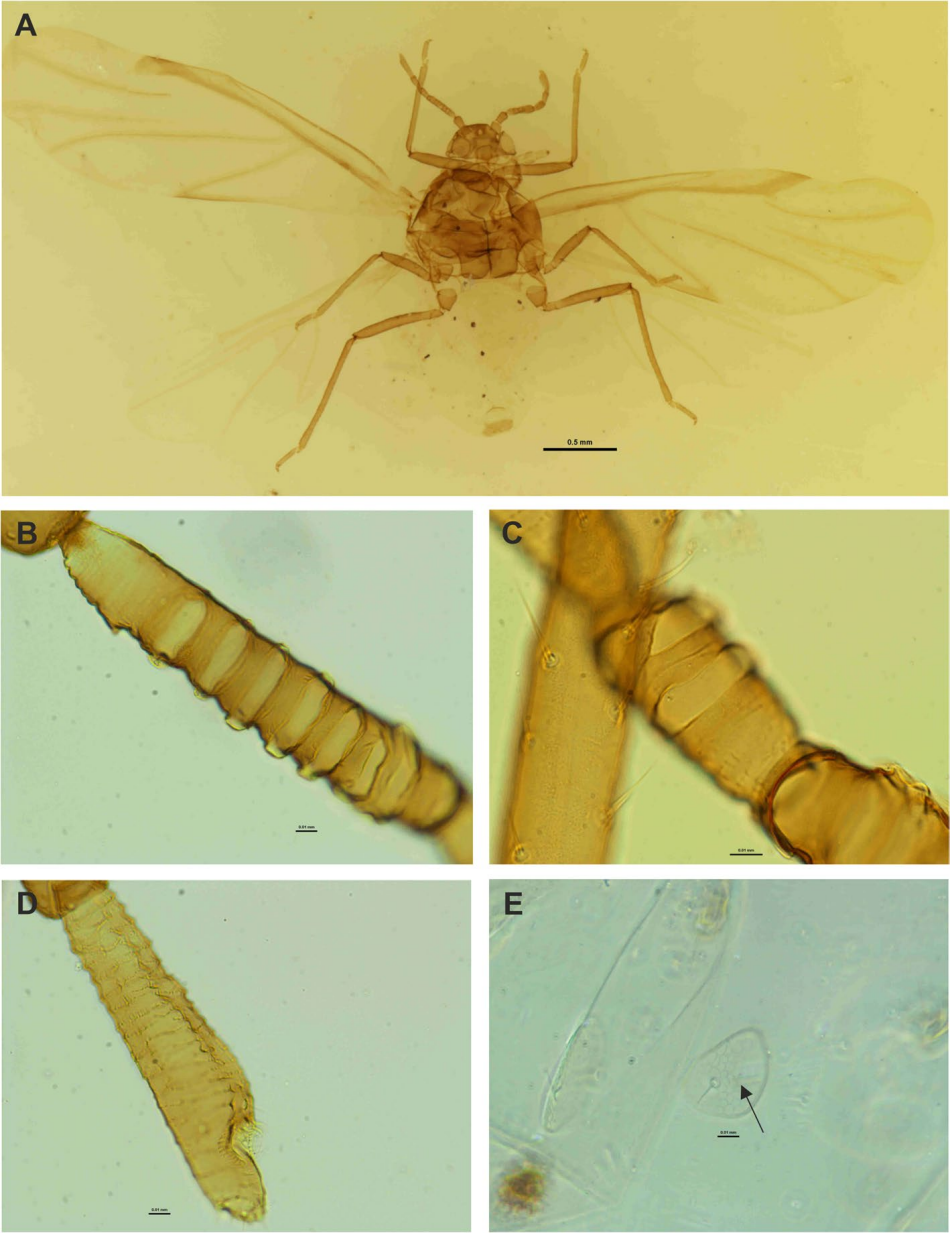
Light microscopy (LM) of the general morphology of *Pemphigus populiglobuli* secondary generation detected in Svalbard. **A** Winged viviparous female (sexupara) collected in Fjortendejulibukta bird cliffs. Antennal segments III **B** and **C** IV with visible large, transverse oval secondary rhinaria. **D** Base of the antennal segment VI with one major and 1–2 poorly visible accessory rhinaria with ciliated cuticle edges. **E** Wax gland plate of marginal abdominal tergite VI (arrow)

Color in life: unknown.

Pigmentation of cleared specimens on slides: head, thorax, antennae, mouthparts, wing veins and legs brown, subgenital and anal plates dusky, abdomen pale (Fig. 7A).

Morphometric characters: body egg-shaped, head not fused with prothorax. Frons flat. Median margin of head with one pair of very short, pointed setae. Compound eyes with triommatidia and ocelli present (Fig. 7A). Antennae 6-segmented, 0.28× body length. Antennal segments II, IV and V of similar length; antennal segment III the longest; processus terminalis very short, 0.31 × base; other antennal ratios: VI:III 0.77, V:III 0.41, IV:III 0.36. Antennae covered with pointed, short, colourless setae that are never longer than the basal articular diameter of antennal segment III. ANT I with 1–2 setae, ANT II with 4–5 setae, ANT III with 6–7 setae, ANT IV with 0–1 setae, ANT V with 1 seta, base of antennal segment VI with two setae, processus terminalis with six apical setae. ANT III with 7–8 (Fig. 7B), ANT IV with 2–3 (Fig. 7C) rather large, transverse oval secondary rhinaria. Apical part of antennal segment V with one primary rhinarium. Base of antennal segment VI with one primary and 1–2 poorly visible accessory rhinaria with ciliated cuticle edges (Fig. 7D). Rostrum reaching to middle coxae. Ultimate rostral segment with a broadly rounded apex, with three pairs of primary setae and lack of accessory setae, 0.41 × antennal segment III, 0.45 × second segment of hind tarsus. Legs normal, first tarsal segments with 3:3:2 setae. Forewings with a single, unbranched media. Subgenital plate broadly rounded. Cauda slightly visible. Siphuncular pores absent. Wax gland plates small, with a small number of glandular pores, present on mesonothum as well as marginal abdominal tergites V-VII (Fig. 7E) and as a larger, single, transverse plate on abdominal tergite VIII.

### Biology

**Fig. 8:**
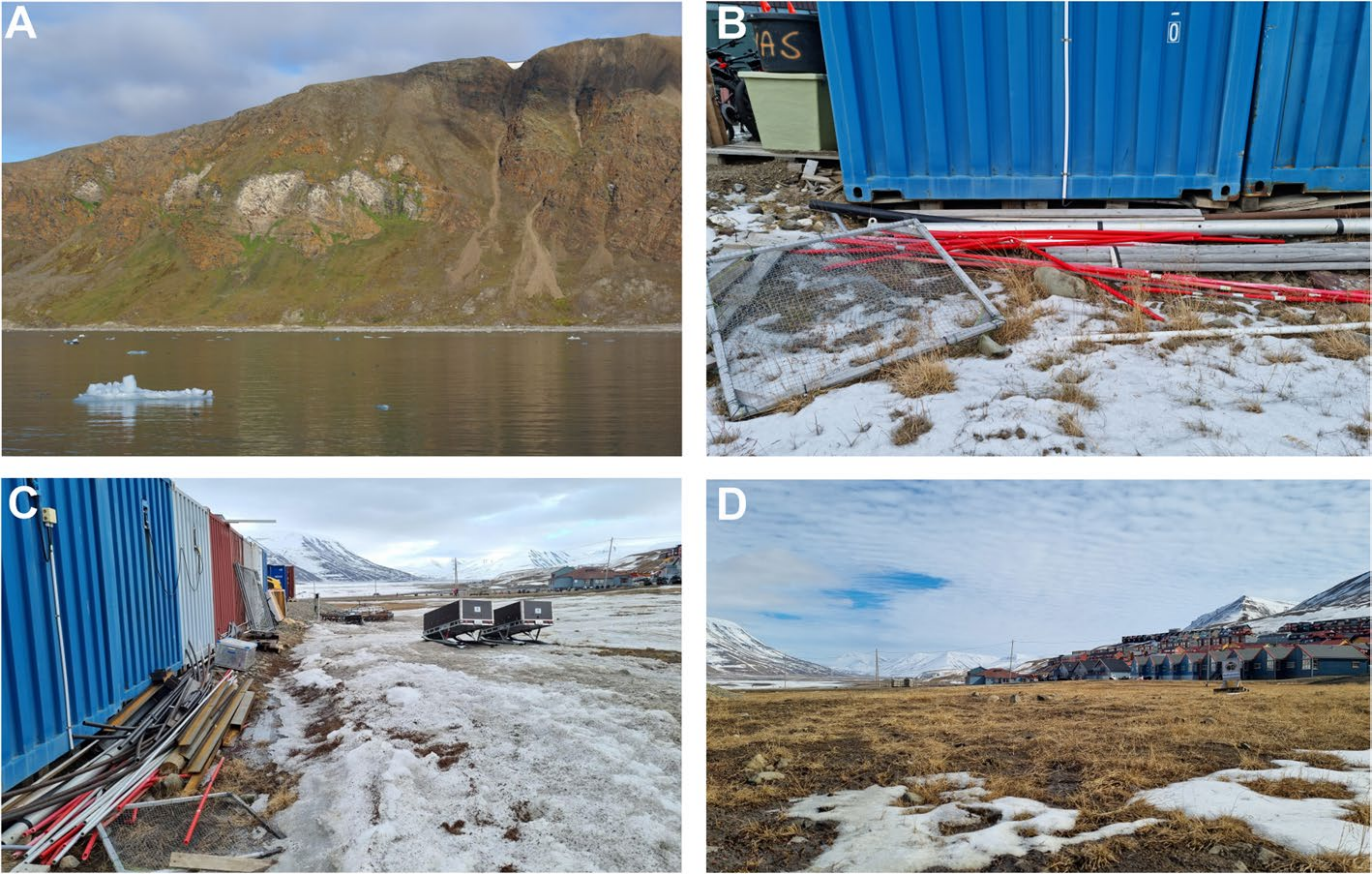
Collection sites of *Pemphigus populiglobuli* secondary generation detected in Svalbard. **A** the lower section of the Fjortendejulibukta bird cliffs (summer population). Area behind the University Centre in Svalbard (UNIS) in Longyearbyen **B** in September 2023, **C** in the beginning of May 2024, **D** at the end of May 2024 (hibernating population)

The waxy colonies of wingless viviparous females and their progeny were mainly found on the grass roots of *Poa* sp., a common grass genus in the Arctic environment. Thor [14] documented the initial observation of these aphids beneath boards within grass and moss, near Barentsburg. In the years 2007–2014, S.J. Coulson collected these aphids from beneath flat stones in the lower section of the Fjortendejulibukta bird cliffs, where the slope angle decreases (Fig. 8A). The stones where these aphids were found were of a specific type; they were not excessively large and were partially submerged in the organic soil, rather than resting on the grass (Fig. 2A). In the years 2007–2009, the colony included numerous individuals. In 2008, a colony of over 100 individuals, including both adults and immatures, was observed under a single stone. The aphids were densely coated with white wax, which also extended to cover the nearby soil surface and remnants of the host plant crushed by the stone. Within the wax secretion, aphid excretion - honeydew droplets coated with wax, forming liquid ‘marbles’, were also visible (Fig. 2B). In 2011, only a very small number of individuals were observed, and there was minimal wax present, whereas in 2014, despite intensive searching, no aphids were found on this location. In 2009, the aphids were collected along with soil samples from the terraced moss tundra at the Diskobukta sampling site on Edgeøya island. In Longyearbyen, the aphids were collected behind a disused cattle shed, initially by G. Søli, and subsequently by S.J. Coulson until 2021. All these observations were conducted between June and August. The earliest observation occurred on 29 June, while the latest was recorded on 25 August. In 2023, directly behind the blue container laboratory number 2 at UNIS (Fig. 8B), S.J. Coulson observed these aphids much later, on 27 September and 16 October. Despite the ground being partly frozen, living specimens were still collected from a fairly numerous colony. As weather conditions are critical for the survival of overwintering soil fauna, including aphids, this location was monitored thorough the entire 2023/2024 winter season. In April, the ground was still well frozen and under snow with a rapid melt at the end of April. However, in the beginning of May it was again below 0 °C and the ground was still frozen (Fig. 8C). At the end of May this area was largely free of snow (Fig. 8D) and soil extractions revealed living specimens of aphids. Their distribution was slightly clumped; some tussocks had many aphids, while others have none. Finally, the monitored population undoubtedly overwintered successfully in situ. Thus, like some other representatives of the *Pemphigus* genus, the secondary generation of this species overwinters as an anholocyclic population, hibernating on grass roots, mainly *Poa* sp., in sheltered microhabitats, such as beneath stones or where a winter snow cover accumulates.

**Fig. 9:**
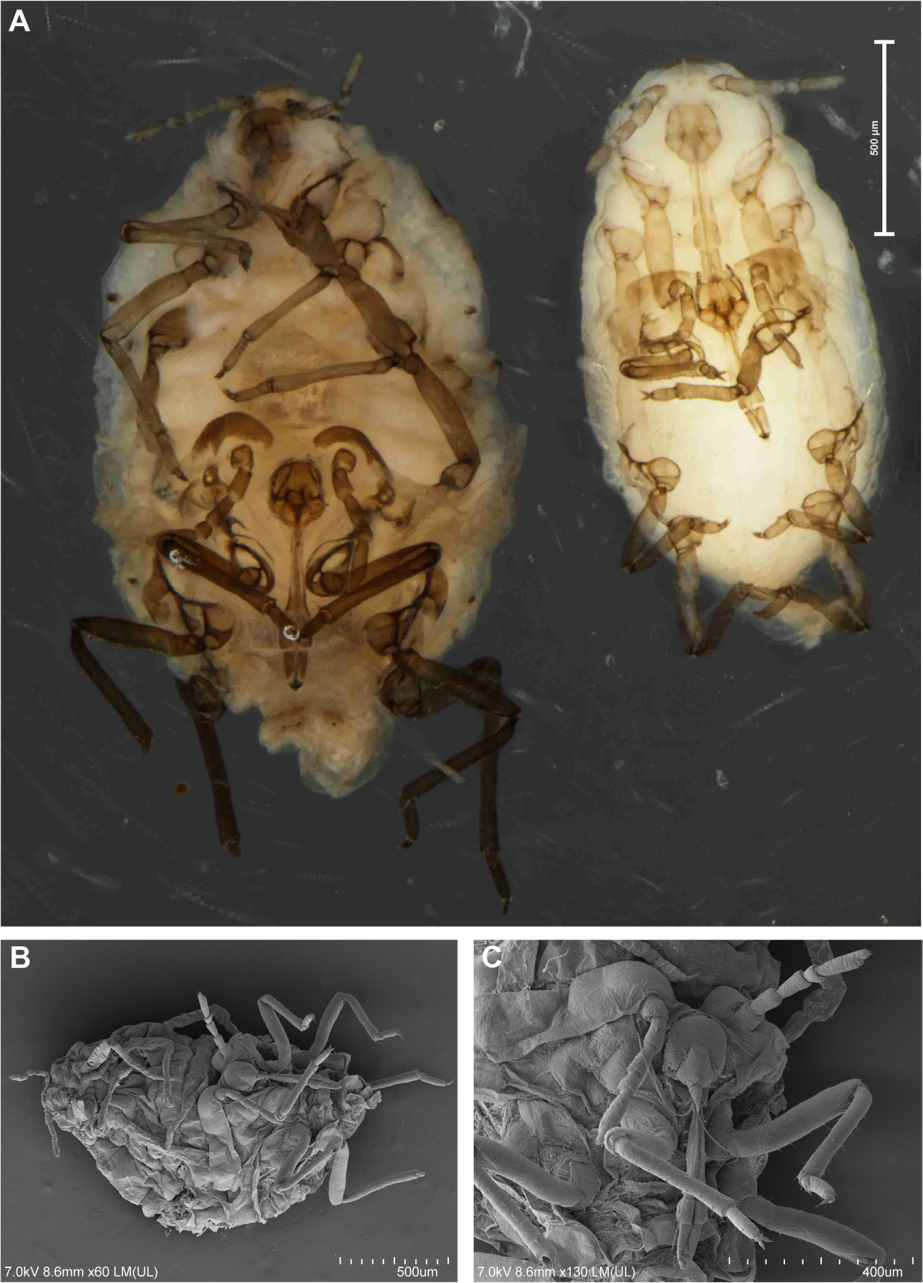
Morphological aberrations of *Pemphigus populiglobuli* secondary generation detected in Svalbard. **A–C** A juvenile individual emerged from the abdomen of an adult wingless viviparous female, displaying antennae and legs extending outside its body, along with a functional rostrum (A-LM, B–C SEM); all collected in Longyearbyen (summer population)

Detailed analysis of the fresh material collected in July 2021 (Longyearbyen sampling site), revealed two adult specimens showing morphological aberrations. A juvenile individual emerged from the abdomen of an adult viviparous female, displaying antennae and legs extending outside its body, along with a functional rostrum, that was approximately twice as long as in other individuals (Fig. 9A–C).

### Remarks

**Fig. 10:**
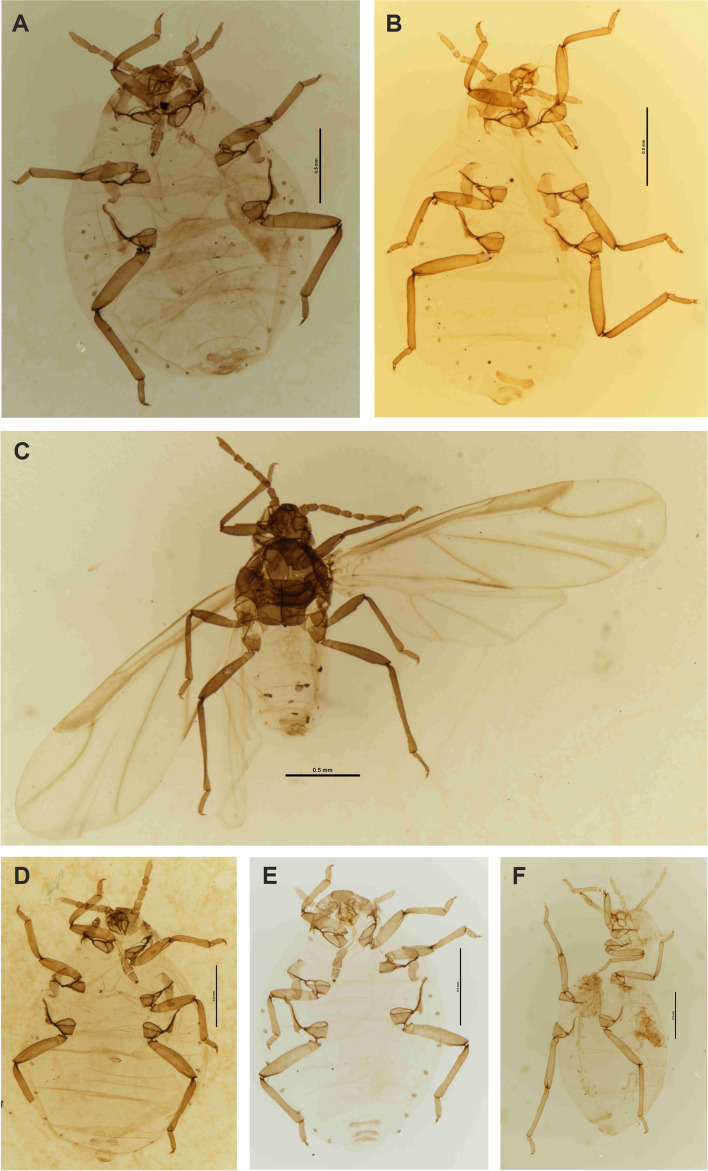
Slide-mounted *Pemphigus populiglobuli* secondary generation, previously identified as *P. groenlandicus* or *P. groenlandicus* subsp. *crassicornis*. **A** wingless viviparous female from Greenland, **B** wingless viviparous female from Iceland, **C** winged viviparous female (sexupara) from Greenland, **D** wingless viviparous female from Greenland, **E** wingless viviparous female from Spain, **F** wingless viviparous female from Sweden (A–C marked as *P. groenlandicus*, D–F marked as *P. groenlandicus* subsp. *crassicornis*). Light microscopy (LM)

The Greenland and Iceland specimens identified as *Pemphigus groenlandicus* analyzed in this study show no substantial morphometric differences compared to the Svalbard population (Table S2, Fig. 10A–B). Despite the absence of fresh material for comparative molecular analysis (in Greenland, these aphids were collected in the 1950s, whereas in Iceland they were collected in the 1970s), the morphological similarities confirm that the Greenland, Iceland and the Svalbard populations are the same species, as mentioned by Hille Ris Lambers [15, 18]. This finding highlights the taxonomic consistency across these geographically separated Arctic populations.

Similarly, no statistically significant differences were found for the subspecies *crassicornis* (Fig. 10D). Hille Ris Lambers designated this subspecies on the basis of its slightly shorter processus terminalis and the different shape of its subgenital plate [15]. However, in the material analyzed here, the measurements of the antennal segments differed only slightly (Table S2), as did the shape of the subgenital plate (Fig. 2E–F). When comparing sexuparae, the main distinguishing features were the length of antennal segment III and the processus terminalis, along with the number of secondary rhinaria on antennal segment III. According to Hille Ris Lambers [18], *Pemphigus groenlandicus* typically has 4–6 secondary rhinaria (Fig. 10C), while *Pemphigus groenlandicus* subsp. *crassicornis* has 3–6. In contrast, one observed sexupara from the Svalbard population displayed seven and eight secondary rhinaria on antennal segments III, whereas two sexuparae from Iceland 4–6 secondary rhinaria on antennal segments III. The number of secondary rhinaria on aphid antennae is highly variable, particularly in winged forms. Therefore, with limited comparative material (single observations of sexuparae in every location), it cannot be reliably used as a diagnostic feature.

The populations from Greenland, Iceland, and Svalbard mentioned earlier share a common ecological niche—they were all found inhabiting grass roots. These biological data strongly support our conclusions regarding the synonymization of *Pemphigus groenlandicus* and its subspecies with *Pemphigus populiglobuli*.

In addition to the Arctic region, aphids of the genus *Pemphigus* associated with grass roots have also been found in Spain and Sweden (MNHN slides). These were identified by Remaudière as *P. groenlandicus* subsp. *crassicornis* [41] and re-described by Pérez Hidalgo and Nieto Nafría [42]. Analysis of this material indicates a high morphometric similarity to the Arctic population, suggesting a strong connection with geographical variability. Specifically, populations from the southern European continent exhibit smaller size of body and antennal segments, contrasting with larger sizes observed in populations from the northern part of the continent. Moreover, specimens from Sweden are characterized by six-segmented antennae, comparing to the remaining samples of this species (Table S2, Fig. 10E–F). The consistent presence of this association across different geographical populations strengthens the argument for considering these to be a single species.

### Distribution

**Fig. 11:**
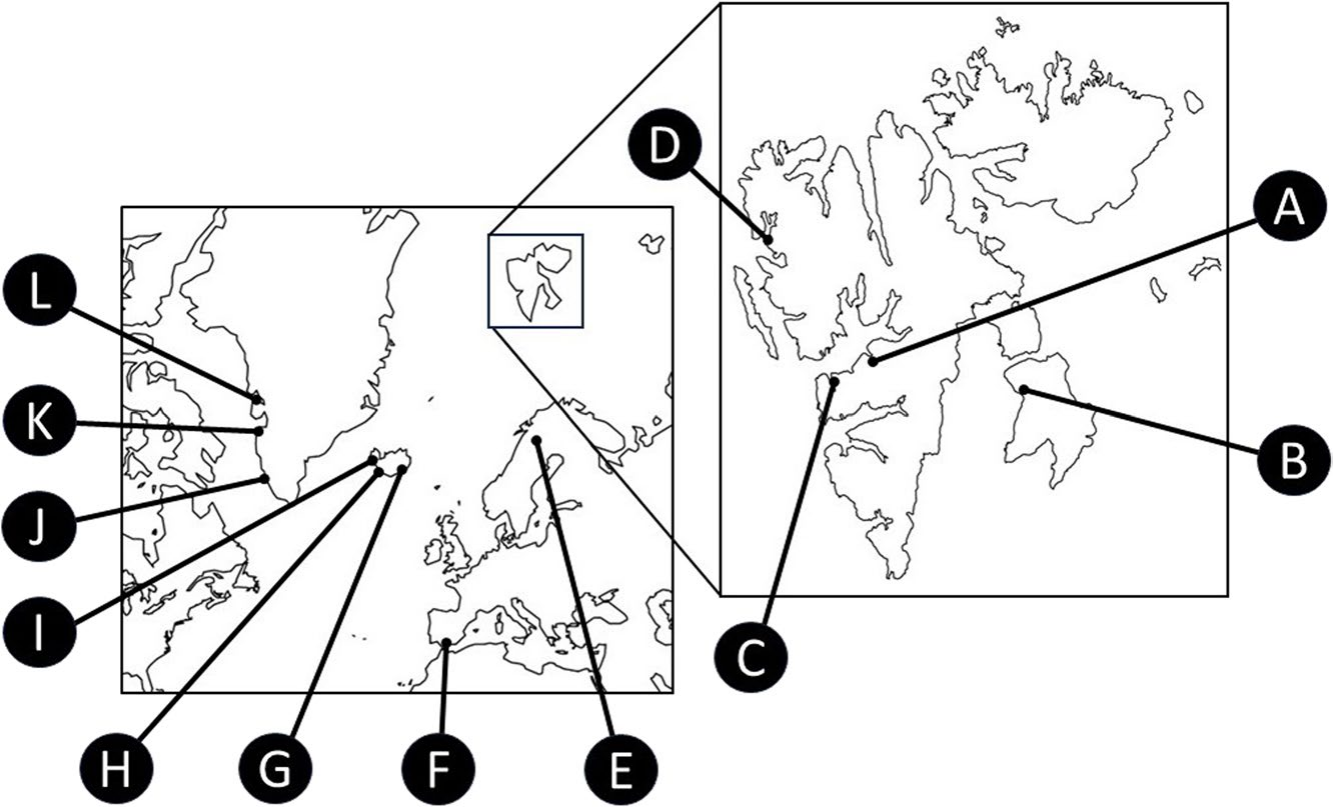
Distribution of *Pemphigus populiglobuli* secondary generation. **A** Longyearbyen (the Svalbard archipelago), **B** Diskobukta, Edgeøya (Svalbard), **C** Barentsburg (Svalbard), **D** Fjortendejulibukta (Svalbard), **E** Abisko National Park (Sweden), **F** Sierra Nevada National Park (Spain), **G** Höfn (Iceland), **H** Vikurbakki Field Station (Iceland), **I** Akureyri (Iceland), **J** Nuuk (Greenland), **K** Aasiaat (Greenland), **L** Uummannaq fiord (Greenland)

Due to its cryptic lifestyle, the secondary generation of *Pemphigus populiglobuli* has been only observed in several scattered locations within the Svalbard archipelago: from the area of ​​Barentsburg (78.04° N 14.22° E) [14], Longyearbyen (78.217° N 15.616° E) and the sampling site Fjortendejulibukta (79.126° N 11.897° E), on Spitsbergen island as well as at the Diskobukta sampling site (77.967° N 21.399° E) on Edgeøya. These sites are situated in the regions of Isfjorden, Krossfjorden, and Storfjorden, respectively. (Fig. 11A–D).

In Greenland it is known from in the innermost part of the Uummannaq fiord (70.403° N 52.752° W) [16], Nuuk, Kapisigdlit (64.261° N 50.166° W) and Aasiaat (Egedesminde) (68.422° N 52.513° W) [15] (Fig. 11J-L).

In Iceland the species has been collected in the vicinity of Akureyri (65.405° N 18.526° W) [17], (NHMUK slides), Höfn (64.151° N 15.123° W) [43] and Iceland Vikurbakki Field Station (64.632° N 21.501° W) [NHMUK slides] (Fig. 11G–I).

Outside these localities, the species has also been found in continental European subarctic area, in Abisko National Park in Sweden (MNHN slides) (Fig. 11E). It was also found in Sierra Nevada National Park in Spain at an altitude of 2,400 m above sea level [42] (Fig. 11F).

## Discussion

### Species identification – DNA barcoding and endosymbiont profile

The COI Maximum Likelihood tree suggests that *Pemphigus* specimens collected from Svalbard are not secondary generations of *Pemphigus bursarius* (Linnaeus) or *Pemphigus borealis* Tullgren, nor their anholocyclic forms, as had been suggested in studies that first identified specimens from *P. groenlandicus* (material from Svalbard [14]; material from Iceland [17]; material from Greenland [15]). This exclusion is also supported by the established trophic interactions of the secondary generation of the mentioned species. Specifically, for *P. borealis*, it is associated with the roots of *Bidens* spp., whereas for *P. bursarius*, it involves the roots of various species within the Compositae family [22]. Our molecular analyses rather suggest that it is the secondary generation from *P. populiglobuli* Fitch (the widely spread Nearctic poplar bullet gall aphid), associated with grass roots, as all the Svalbard specimens are clustered with *P. populiglobuli* specimens. The Svalbard specimens might have lost primary host associations and live all year round on grass roots. To date, this generation has not been reported in *P. populiglobuli*.

Overall, our study reveals that species identification can be difficult in the *Pemphigus* genus (Additional file 1); there were many unidentified specimens in BOLD database, the clade formed by the *P. populiglobuli*,* P. groenlandicus*,* P. monophagus* Maxson and our specimens also included many unidentified specimens for which host associations were not indicated (Additional file 4). This suggests that species delimitation may on some occasions have been based erroneously on host associations or gall morphology. This statement is likely supported by the inclusion of samples identified as *P. monophagus* in the *P. populiglobuli*/*Pemphigus* from the Svalbard clade (Fig. 1). While *P. monophagus* and *P. populiglobuli* have a similar range of occurrence in North America and share the same relationships with the primary hosts (*Populus angustifolia*,* P. balsamifera*,* P. trichocarpa*), they differ significantly in their biology. *Pemphigus monophagus* does not have a secondary host, completing its entire life cycle on the primary *Populus* host [22]. According to molecular study of Foottit et al. [24], several taxa, including *P. betae* Doane, *P. populivenae* Fitch, and, the third undescribed species of *Pemphigus*, form morphologically indistinguishable galls on the leaf blades of species and hybrids of *Populus* section Tacamahaca, often on the same tree. Gall morphology is frequently used to identify *Pemphigus* species; however, this practice may be unreliable for taxa associated with the same host tree. The limitations of gall morphology, coupled with the need for more comprehensive molecular studies, highlight the necessity of integrating detailed genetic and ecological data to refine species identification methods within the *Pemphigus* genus, and to clarify species relationships and boundaries.

Concerning associations with endosymbionts, the results of the present study show that our three specimens do not host any known facultative symbionts. The occurrence in our sequencing of a *Pseudomonas*, closely related to *Pseudomonas prosekii*, a cold tolerant bacteria that has been described from the soil in Antarctic [44], probably stems from environmental contamination of our specimens with bacteria found in their habitat, as *Pseudomonas* is prevalent in Svalbard’s permafrost soils [45].

### Species identification – morphology

According to Blackman et al. [46], the identification of *Pemphigus* wingless viviparous females found on the roots of their secondary hosts is very difficult, if not impossible, when only morphological characters are used. Thus, these species are more reliably separated based on biological than on morphological characters [47]. Moreover, this secondary generation varies little between species, but can exhibit substantial intraspecific variation due to environmental factors, particularly when colonies overwinter.

In our study, we examined the Greenland and Iceland specimens identified as *Pemphigus groenlandicus* and compared them morphometrically with the Svalbard specimens. Despite the absence of fresh material from all known locations, which limits our ability to fully validate morphological findings with genetic data, the morphological similarities across these Arctic populations confirm their classification as the same species, supporting Hille Ris Lambers’ conclusions [15, 18]. Additionally, no significant differences were observed for the subspecies *P. groenlandicus* subsp. *crassicornis*. Variations in the antennal segments length and subgenital plate shape were minor, and did not support distinct subspecies status. The number of secondary rhinaria of sexuparae, a highly variable feature, was not reliable for distinguishing the taxa. Furthermore, populations from Greenland, Iceland, and Svalbard share a common ecological niche, inhabiting grass roots, reinforcing the synonymization of *P. groenlandicus* and its subspecies with *Pemphigus populiglobuli*. Specimens from Spain and Sweden also exhibit high morphometric similarity to the Arctic populations, suggesting a connection with geographical variability. The unique grass root association across these populations supports the conclusion that they represent a single species. An important ecological feature of these aphids is the secretion of a waxy material by individual aphids which gives their subterranean colonies a moldy appearance. This waxy substance repels moisture and may protect the aphids from their own honeydew due to the absence of a cauda or ant attendance, a behavior also observed in other species such as *P. bursarius* (Linnaeus) [48] and *P. betae* Doane [49].

### Biology

Insects that inhabit Arctic regions face extreme environmental conditions, including cold temperatures, short growing seasons, and limited food resources [50, 51]. Despite these challenges, various insect species have developed strategies to survive and thrive in Arctic conditions. Some Arctic insects are capable of supercooling, a process in which they lower their body temperatures below the freezing point of their bodily fluids without actually freezing. This helps such species to avoid ice formation within their bodies, preventing cell damage. Insects may also produce cryoprotectants, such as glycerol or antifreeze proteins, which help lower the freezing point of their bodily fluids and protect them from ice crystal formation. Synchronization with seasonal conditions, diapause at various life stages and behaviors to find suitable conditions for feeding, reproduction, and development are another common strategies employed by Arctic insects [12, 52–54]. Although the physiological adaptations of Svalbard *Pemphigus* to Arctic conditions are outside of the scope of the current work, they are undoubtedly capable of overwintering as active forms, i.e. adults and nymphs (anholocyclic species/clones), as was observed in the Longyearbyen UNIS sampling site in the season 2023/2024. Several years of observations at the Fjortendejulibukta site also demonstrate the survival of these aphids. However, with longer-term observation, these locations may prove to be ephemeral, as indicated by the small number of individuals found in 2011 and the complete absence of colonies in 2014. Moreover, extreme weather events, such as warm spells and heavy rain-on-snow events, are becoming more frequent in the Svalbard archipelago during the polar night, additionally influencing Arctic organism survival [55]. Sheltered microhabitats, such as beneath rocks, in roots vegetation, provide insulation and protection from year-round wind and extreme temperatures. Undoubtedly, the secondary generation of *P. populiglobuli* Fitch is an example of terrestrial microarthropod fauna that overwinters in situ in soil and vegetation. Snow depth and winter air temperatures regulate soil microarthropod populations locally, impacting Arctic soil ecosystems [56]. Thus, reproduction success and survival rates of subterranean aphids are likely due to high variation in microenvironments. This has also been reported for aphids inhabiting similar microhabitats as moss layers [57]. Moran and Whitham [58] observed that soil temperature alone did not determine the reduction of the life cycle in *P. betae* Doane; it was also strongly affected by elevation and the associated availability of host plants. Phillips et al. [59] further supported this observation, suggesting that factors such as soil moisture or humidity may play a more crucial role in the survival and overwintering strategies of *P. bursarius* (Linnaeus), the species whose hibernating morphs are physiologically distinct from its summer morphs.

Our molecular investigation suggests that the *Pemphigus* population in Svalbard is a secondary generation of *P. populiglobuli*. This species is exclusively identified through the gall-inducing generation found on the primary hosts—balsam poplars (*Populus*, section Tacamahaca)—in its Nearctic range of distribution. The absence of the primary host tree species results in aphids being anholocyclic in Arctic conditions. However, the capacity for host-alternation has not been entirely lost, as indicated by the observation of a winged viviparous female (sexupara), at the Fjortendejulibukta sampling site. This morph was gathered in early August 2009, marking the sole observation of this kind throughout the entirety of the research period.

### Biogeography

Although there is some overlap in their geographical distribution, the known primary hosts of *Pemphigus populiglobuli* Fitch i.e. *Populus angustifolia*,* P. balsamifera*, and *P. trichocarpa* occupy relatively distinct regions in North America. For example, narrowleaf cottonwood is found primarily in the central and southern Rocky Mountain regions, the balsam poplar is found in the northern Rockies into Canada and extending east to the Great Lakes region, and the black cottonwood has a range stretching from Alaska, through western Canada, and primarily into the northwestern United States, but also sporadically down as far south as Baja, California [49]. Despite poplars’ distribution coinciding with the availability of potential secondary hosts of *P. populiglobuli*, for example in Canada’s grasslands [60], only the gall-inducing generation is known to be widely distributed in North America [22]. According to our research, the secondary generation of *P. populiglobuli*, mostly described as *P. groenlandicus*, has been found in isolated and remote sites on Arctic islands and mountainous regions in Europe. Currently, due to the harsh climatic conditions, no native poplar species are found in the high Arctic regions of Greenland or the Svalbard archipelago. However, ancient environmental DNA (eDNA) records, such as those from the Kap København Formation in North Greenland, indicate that poplar species related to modern *P. trichocarpa* and *P. balsamifera* were present around two million years ago, in the early Pleistocene, during periods when the climate was warmer [61]. In contrast, black cottonwood (*P. trichocarpa*) was first introduced to Iceland in 1944 from the Kenai Peninsula, Alaska, and it has become an important urban tree in Iceland, widely planted in forestry since the 1990s [62]. Thus, there is a potentially available host plant on which aphids can complete their life cycle. The presence of two winged females on a black poplar in Iceland (NHMUK slide) supports this hypothesis. The label of the slide suggests that this was a successful experimental transfer of *Pemphigus* individuals from grass to their primary host, poplar. Thus, the observation of winged viviparous females (sexuparae) in all Arctic locations proves that the ability for host alternation has not been entirely lost in this species, even if the primary host plant is absent, as in the case of Svalbard or Greenland.

When investigating the occurrence of *P. populiglobuli* outside Arctic or subarctic islands, the distribution in continental Europe can provide valuable insights into the species’ adaptability and environmental shifts. Abisko National Park is located in the Scandinavian Mountains in northern Sweden, just north of the Arctic Circle. It is known for its high-latitude summits, which bring a subarctic climate with cold winters and cool summers [63]. Sierra Nevada National Park is situated in southern Spain and is characterized by its Mediterranean climate, with warm summers and relatively mild winters, albeit with significant snowfall at higher elevations. Despite its southern location, Sierra Nevada has unique microclimates at higher altitudes that support species typically found in colder climates [64]. The presence of *P. populiglobuli* in Abisko and Sierra Nevada National Parks highlights the complex interplay of climate, geography, and historical factors that allow this species to thrive outside their usual ranges. In Abisko, the naturally cold climate supports such species, while in Sierra Nevada, high-altitude microclimates provide suitable conditions for their survival. These observations underscore the importance of preserving diverse habitats to support species with specific ecological requirements and contribute to our understanding of species distribution in response to climatic changes.

The study of Arctic insects, including aphids, faces significant limitations. The physical environment’s harsh conditions, remote locations, and challenging logistics, coupled with a relatively short history of both classical taxonomic and modern molecular phylogenetic studies, combine to make these investigations particularly challenging. Consequently, we can suspect that due to the cryptic lifestyle of the secondary generation of *Pemphigus populiglobuli*, its distribution may be broader than currently known.

## Conclusions

In summary, study of aphids within the genus *Pemphigus* presents significant challenges in species identification due to morphological similarities and diverse host plant associations. These aphids demonstrate complex life cycles involving both primary and secondary host plants, with adaptations including root-feeding strategies.

Recent samples from Svalbard of *Pemphigus* provided crucial specimens for molecular and morphological analysis, yielding insights into the taxonomic relationships and morphometric characteristics of grass-feeding *Pemphigus* populations across Arctic and European locations. Molecular analyses indicated that the Svalbard specimens are not a distinct species but rather a secondary generation of *Pemphigus populiglobuli*, adapted to year-round association with grass roots. Comparative studies involving samples from Greenland, Iceland, Sweden, and Spain showed significant morphometric similarities among geographically distant populations, suggesting synonymization of *P. groenlandicus* and its subspecies with *P. populiglobuli*. More extensive molecular analyses are necessary to clarify the relationships and boundaries between closely related *Pemphigus* species, especially given the potential for morphological overlap and the challenges in host association-based identification.

## Supplementary Information

Supplementary Material 1. AdditionnalFile1.newick (https://itol.embl.de/upload.cgi); Tree file in newick format built from ML analysis of all Pemphigus COI sequences present in Bold (at the time of the study) and identified at the species level.

Supplementary Material 2. Table S1. Table of abundance of bacteria across the three Pemphigus specimens sampled in this study resulting from the analysis of 16 S rDNA fragment high-throughput sequencing.

Supplementary Material 3. Table S2. Measurements of Pemphigus populiglobuli secondary generation from all known locations.

Supplementary Material 4. AdditionnalFile4.newick (https://itol.embl.de/upload.cgi); Tree file in newick format built from ML analysis of all Pemphigus COI sequences present in Bold (at the time of the study).

## Data Availability

All data generated or analyzed during this study are included in this published article [and its supplementary information files]. Genetic data are deposited in GenBank, NCBI.
